# Supplementary material for: Eco-evolutionary strategies for relieving carbon limitation under salt stress differ across microbial clades
Source: Nat Commun. 2024 Jul 17;15:6013. doi: 10.1038/s41467-024-50368-z (PMC11255312; doi:10.1038/s41467-024-50368-z)
Supplement: Supplementary file 1 — Supplementary Information [file 41467_2024_50368_MOESM1_ESM.pdf]

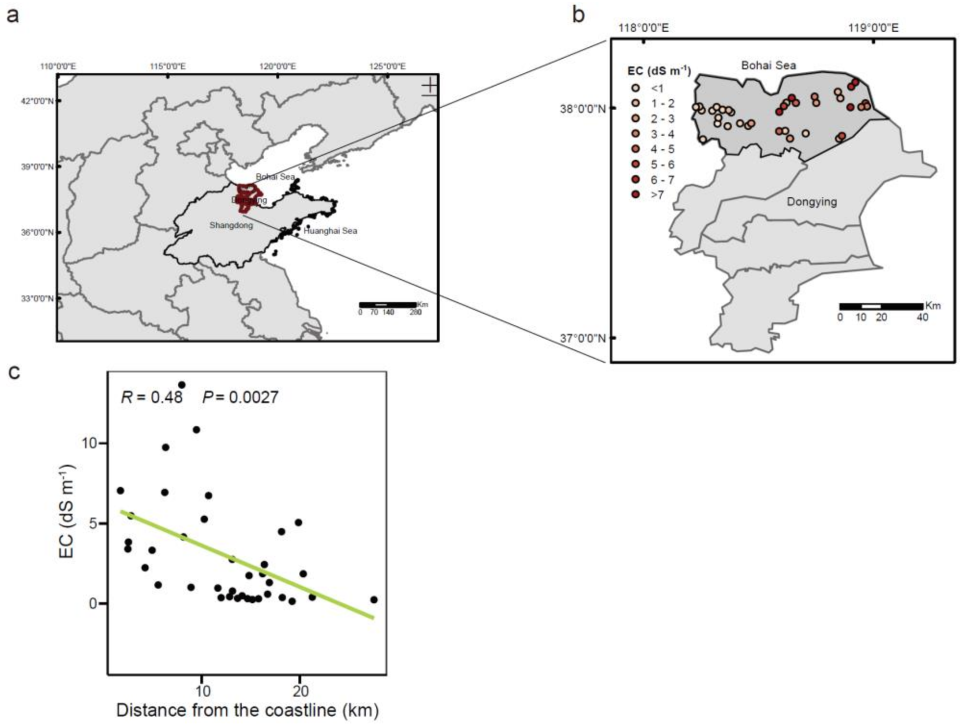


**Fig. S1 Sampling sites for the 37 soil samples and their salinity distribution. a** Map of the area around Shandong Province. **b** Distribution map of sampling sites in Dongying. **c** Ordinary least squares linear regression between electric conductivity (EC) and the distance of the sampling site from the coastline (two-sided, $P < 0.05$ indicates a significant correlation). Near Dist function in ArcGIS 10.7 software was used calculate the shortest distance from the sampling site to the coastline. Source data are provided as a Source Data file.

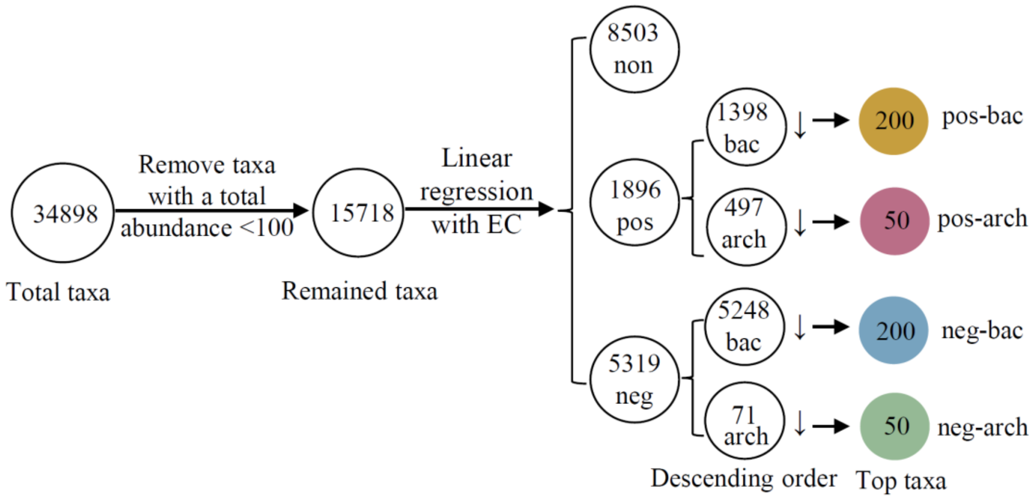


**Fig. S2 Workflow for establishing four response groups.**

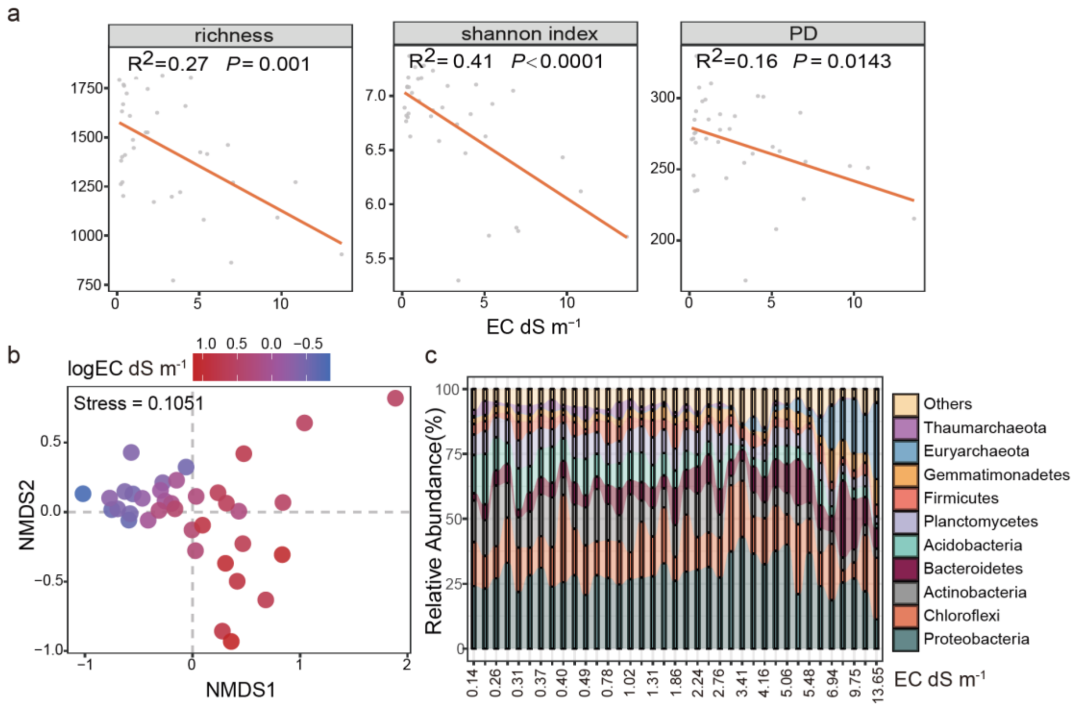


**Fig. S3 Changes in microbial communities across the soil salinity gradients. a** Ordinary least squares linear regression between microbial alpha diversity and electric conductivity (EC) (two-sided, $P < 0.05$ indicates a significant correlation). **b** Nonmetric multidimensional scaling (NMDS) plot of microbial community. **c** Taxa succession of the top 10 abundance at the phylum level. Source data are provided as a Source Data file.

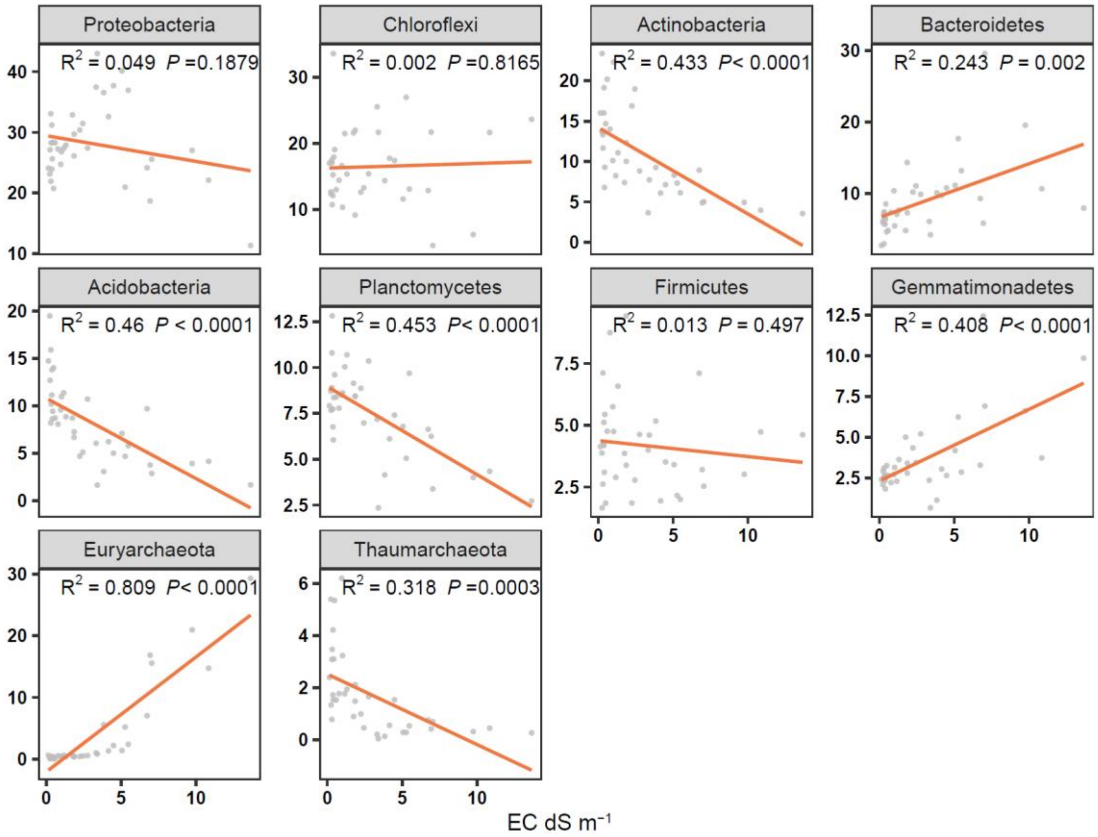


**Fig. S4 Ordinary least squares linear regression between relative abundance of phylum taxa and electric conductivity (EC)** (two-sided, $P < 0.05$ indicates a significant correlation)**.** Source data are provided as a Source Data file.

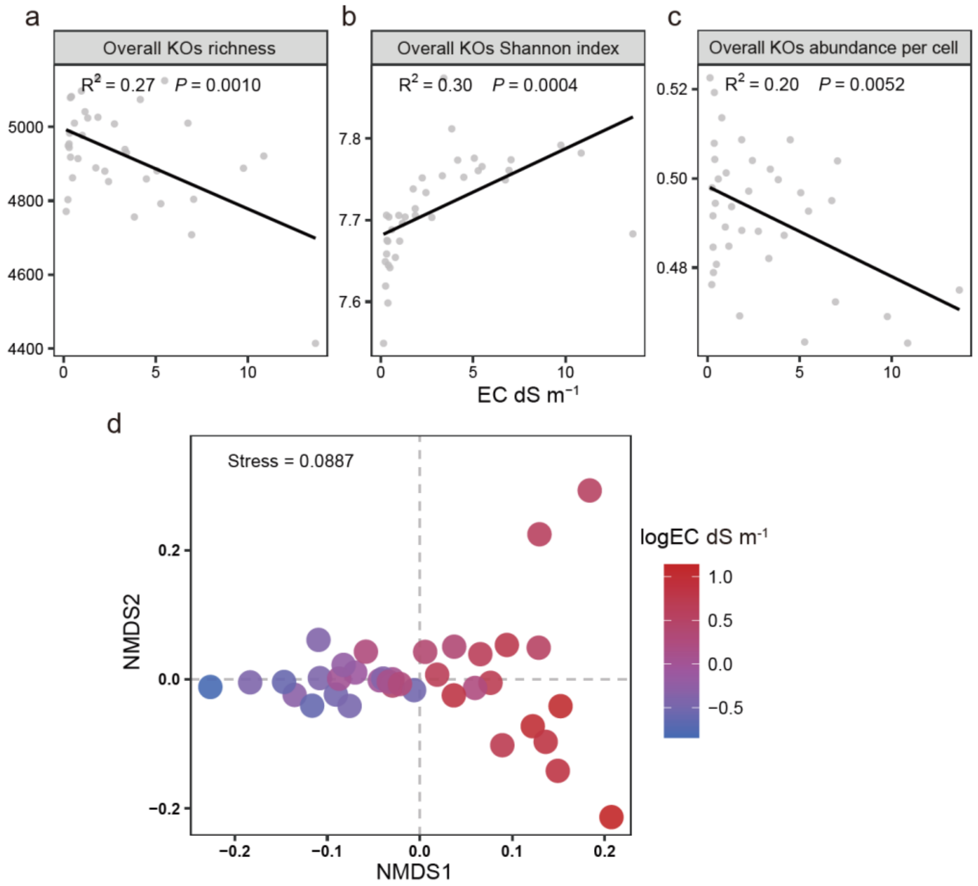


**Fig. S5 Changes in microbial functions of all taxa across the soil salinity gradients. a to c** Linear responses of KOs richness **(a)**, KOs Shannon index **(b)**, and KOs abundance to electric conductivity (EC) **(c)**. *P*-values are calculated by two-sided ordinary least squares linear regression ($P < 0.05$ indicates a significant correlation). d Nonmetric multidimensional scaling (NMDS) plot of KOs. Source data are provided as a Source Data file.

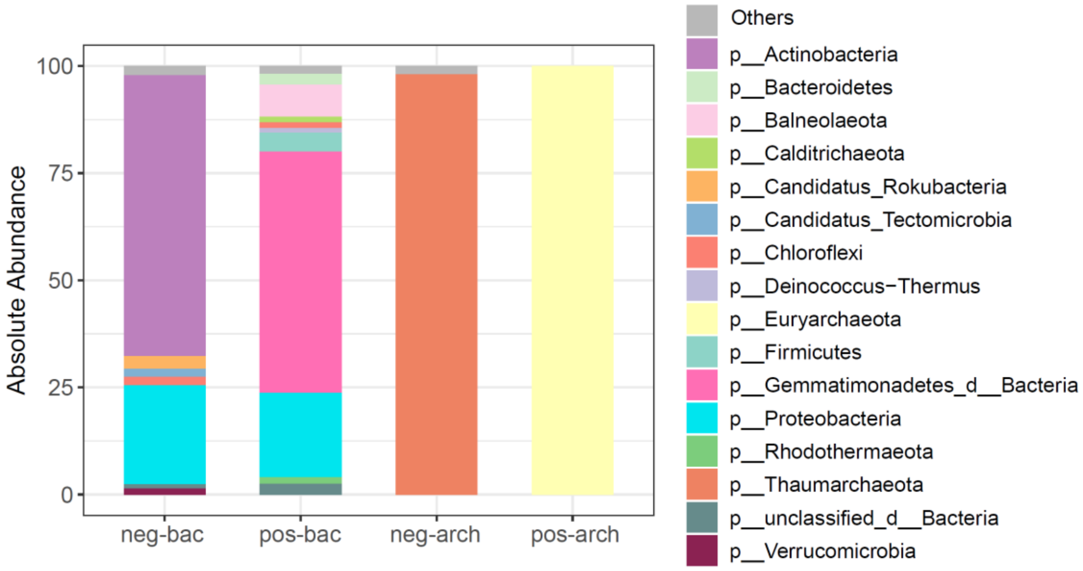


**Fig. S6 Relative abundance of dominant phyla in four response groups.** Source data are provided as a Source Data file.

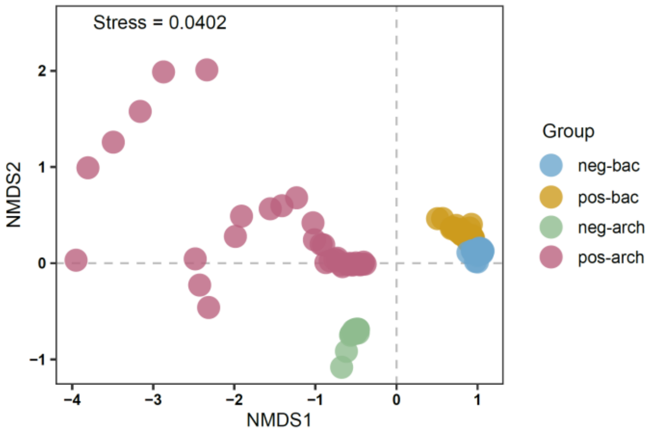


**Fig. S7 Bray-Curtis distance-based nonmetric multidimensional scaling (NMDS) for KOs in four response groups.** Source data are provided as a Source Data file.

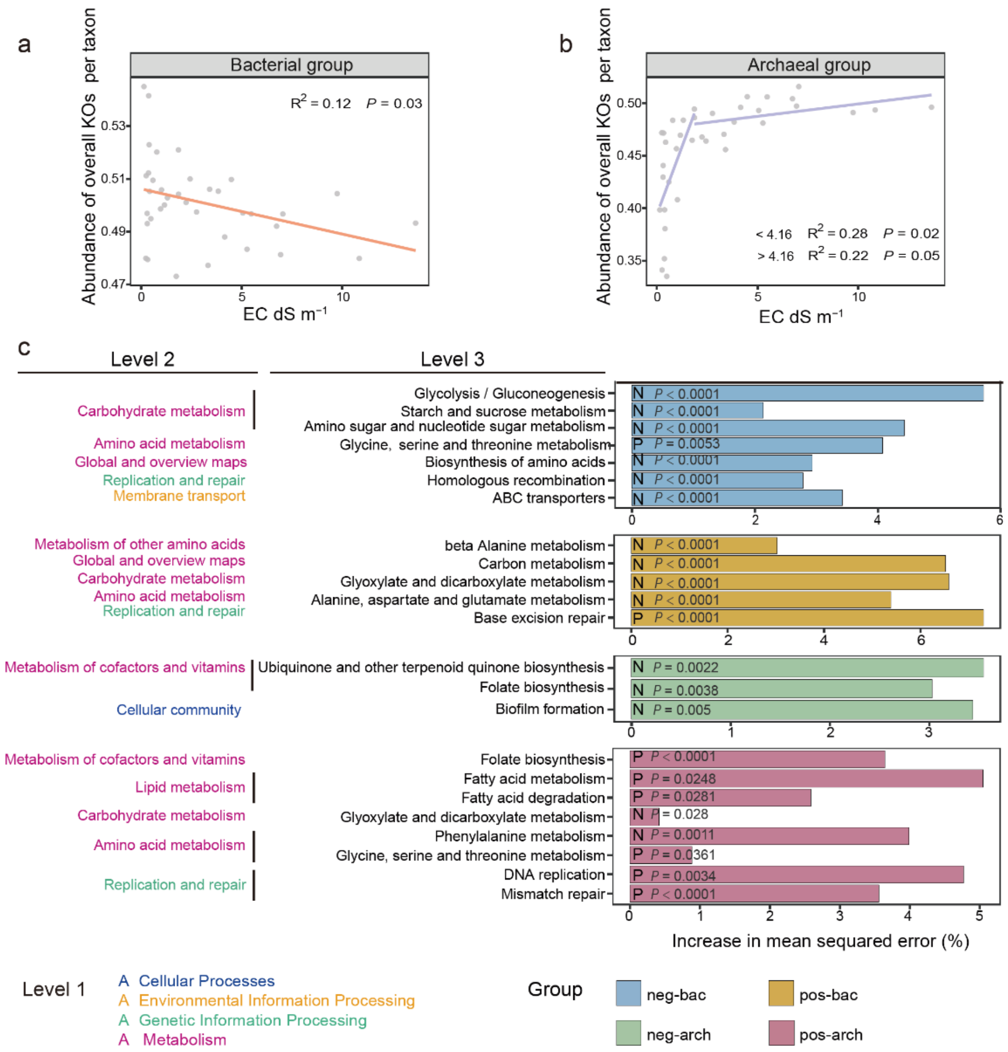


**Fig. S8 Response of functional genes to salinity in each response groups. a to b** Ordinary least squares linear regression between abundance of the overall KOs and EC. **c** KEGG pathways biomarkers at level 3 along the salinity gradients. The letters P and N on the right of the plot represent significant positive and negative correlations between corresponding pathway and EC, respectively. *P*-values are calculated by two-sided ordinary least squares linear regression ($P < 0.05$ indicates a significant correlation). Source data are provided as a Source Data file.

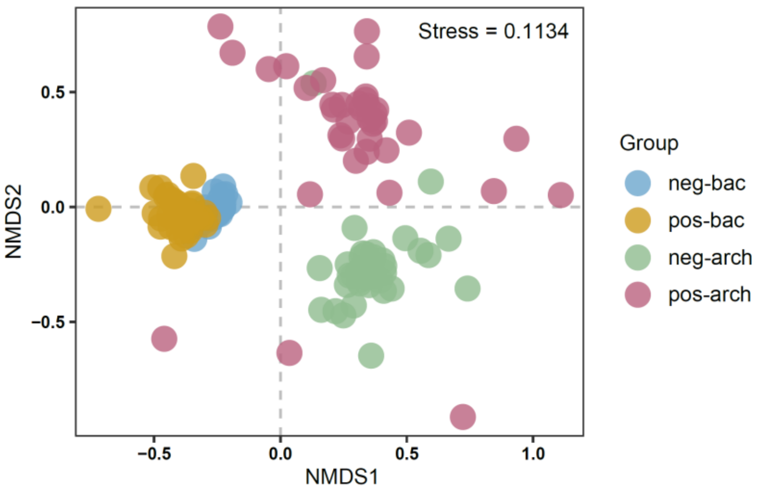


**Fig. S9 Bray-curtis distance-based nonmetric multidimensional scaling (NMDS) for the KOs related to C-acquisition in four response groups.** Source data are provided as a Source Data file.

**Table S1. PERMANOVA analyses of the KOs associated with carbon-harvesting in four response groups**

| Pairwise Test | F. Model | $R^2$ | $P$ |
|---|---|---|---|
| pos-bac vs neg-bac | 27.75 | 0.28 | 0.001 |
| pos-arch vs neg-arch | 36.93 | 0.34 | 0.001 |
| pos-bac vs pos-arch | 77.11 | 0.52 | 0.001 |
| neg-bac vs pos-arch | 61.27 | 0.46 | 0.001 |
| pos-bac vs neg-arch | 183.80 | 0.72 | 0.001 |
| neg-bac vs neg-arch | 168.95 | 0.70 | 0.001 |

Note: $P < 0.05$ indicates a significant correlation. Source data are provided as a Source Data file.

**Table S2. Partial correlation (Two-sided) between edaphic factors and abundance of overall KOs and C-acquisition genes, with EC as the control variable**

| Dependent variable | Independent variable | Control variable | $P$-value |
|---|---|---|---|
| Abundance of overall KOs of bacterial taxa (normalized by taxa abundance) | SOC | EC | 0.122 |
| | DOC | EC | 0.370 |
| | pH | EC | 0.171 |
| | TN | EC | 0.454 |
| | AN | EC | 0.290 |
| | TP | EC | 0.461 |
| Abundance of C-acquisition genes of bacterial taxa (normalized by taxa abundance) | SOC | EC | 0.873 |
| | DOC | EC | 0.963 |
| | pH | EC | 0.344 |
| | TN | EC | 0.088 |
| | AN | EC | 0.052 |
| | TP | EC | 0.353 |
| Abundance of overall KOs of archaeal taxa (normalized by taxa abundance) | SOC | EC | 0.296 |
| | DOC | EC | 0.108 |
| | pH | EC | 0.633 |
| | TN | EC | 0.741 |
| | AN | EC | 0.389 |
| | TP | EC | 0.463 |
| Abundance of C-acquisition genes of archaeal taxa (normalized by taxa abundance) | SOC | EC | 0.490 |
| | DOC | EC | 0.283 |
| | pH | EC | 0.551 |
| | TN | EC | 0.069 |
| | AN | EC | 0.855 |
| | TP | EC | 0.131 |

Note: $P < 0.05$ indicates a significant correlation. Source data are provided as a Source Data file.
