# Supplementary material for: Eco-evolutionary strategies for relieving carbon limitation under salt stress differ across microbial clades
Source: Nat Commun. 2024 Jul 17;15:6013. doi: 10.1038/s41467-024-50368-z (PMC11255312; doi:10.1038/s41467-024-50368-z)
Supplement: Supplementary file 3 — Description of Additional Supplementary Files [file 41467_2024_50368_MOESM3_ESM.pdf]

Description of Additional Supplementary Files

Supplementary Data 1. Information of sampling sites and physicochemical properties of samples.

Supplementary Data 2. Taxa abundance of each sample in four groups.

Supplementary Data 3. Genome size of target taxa.

Supplementary Data 4. KOs related to inorganic carbon-fixation.

Supplementary Data 5. KOs related to salt-resistance.
